# Circadian Rhythm-Related Characteristics and Adiposity in Relation to CAP-Defined Hepatic Steatosis Among Shift and Non-Shift Workers: A Pilot Study

**DOI:** 10.3390/nu18213492

**Published:** 2026-10-23

**Authors:** Neslişah Rakıcıoğlu, Rüveyda Esra Özkalaycı, Canan Altınsoy, Çağlayan Keklikkıran, Eda Kaya, Yusuf Yılmaz

**Affiliations:** 1 Department of Nutrition and Dietetics, Faculty of Health Sciences, Hacettepe University, Ankara 06100, Türkiye; neslisah@hacettepe.edu.tr; 2 Department of Nutrition and Dietetics, Faculty of Health Sciences, Recep Tayyip Erdoğan University, Rize 53350, Türkiye; ruveydaesra.ozkalayci@erdogan.edu.tr (R.E.Ö.); canan.altinsoy@erdogan.edu.tr (C.A.); 3 Department of Gastroenterology, School of Medicine, Recep Tayyip Erdoğan University, Rize 53350, Türkiye; yusuf.yilmaz@erdogan.edu.tr; 4 The Global NASH Council, Washington, DC 20037, USA; eda.kaya@knappschaft-kliniken.de; 5 Department of Medicine, Knappschaft Kliniken University Hospital Bochum, Ruhr University Bochum, 44892 Bochum, Germany; 6 Dijitalpark Teknokent, İstanbul 34788, Türkiye

**Keywords:** hepatic steatosis, controlled attenuation parameter, shift work, social jetlag, eating jetlag, sleep quality, anthropometric measurements, adiposity indices

## Abstract

**Background/Objectives**: Metabolic dysfunction-associated steatotic liver disease (MASLD) is the most prevalent chronic liver disease worldwide and linked to shift work. This study aimed to investigate the associations of circadian rhythm, sleep characteristics, anthropometric measures, and adiposity-related parameters with controlled attenuation parameter (CAP) values, and to assess their independent associations and sex-specific discriminatory performance in identifying hepatic steatosis among shift and non-shift workers. **Methods**: A total of 436 workers (328 men and 108 women), aged 19–64 years, from seven large-scale tea factories in Rize, Türkiye, were included. Social jetlag (SJL) and eating jetlag (EJL) were calculated from workday–free-day differences in sleep and meal timing, respectively, and sleep quality was assessed using the Pittsburgh Sleep Quality Index (PSQI). Multivariable linear regression and sex-specific receiver operating characteristic analyses were performed. **Results**: Mean SJL (*p* = 0.017) and the distribution of SJL categories (*p* = 0.022) differed significantly between female shift and non-shift workers, whereas neither differed in men (*p* = 0.827 and *p* = 0.542, respectively). These comparisons are exploratory because SJL and EJL estimates are not directly comparable across rotating shift assignments or between shift and non-shift workers. CAP values and the prevalence of CAP-defined hepatic steatosis did not differ significantly by shift-work status in either sex. CAP was positively correlated with anthropometric and adiposity-related measures, with the strongest adjusted correlations observed for waist circumference, abdominal volume index (AVI), BMI, and body fat mass (all r_s_ = 0.66, *p* < 0.001). In the fully adjusted regression model, age, BMI, and neck circumference were independently associated with higher CAP values, whereas SJL, EJL, sleep duration, and PSQI total score were not. In sex-specific ROC analyses, waist circumference and AVI showed the highest discriminatory performance in men (both AUC = 0.86), whereas BMI showed the highest discriminatory performance in women (AUC = 0.81). SJL and EJL did not significantly discriminate hepatic steatosis in either sex, while sleep duration showed weak discrimination in women (AUC = 0.63, *q* = 0.042). **Conclusions**: Anthropometric and adiposity-related measures were strongly associated with CAP values and showed good sex-specific discriminatory performance for hepatic steatosis. However, SJL findings, particularly comparisons by shift-work status, should be considered exploratory given the limited comparability of SJL and EJL estimates among rotating shift workers and between shift and non-shift workers. These simple, readily obtainable anthropometric measurements and derived adiposity indices may facilitate preliminary risk stratification for hepatic steatosis in occupational settings.

## 1. Introduction

Shift work is a form of employment that requires individuals to work during time periods that are out of sync with their biological clocks. With globalization and the rise of sectors requiring 24/7 uninterrupted service, shift work has become increasingly widespread. While approximately 10–17% of the global workforce is employed under a shift work system, this percentage ranges from 15–25% in industrialized countries and is particularly prevalent in the healthcare, manufacturing, transportation, and security sectors [1]. Night shifts and rotating shifts cause chronic misalignment between the organism’s endogenous circadian rhythm and environmental timers such as the light–dark cycle, sleep–wake patterns, and feeding schedules. Social jet lag (SJL), which reflects the difference in sleep–wake timing between workdays and free days, places a burden on the biological clock due to irregular work hours, sleep restriction, and varying rest periods [2,3]. In individuals experiencing SJL, eating jet lag (EJL) is observed as a result of variations in meal timing between workdays and free days [4].

Shift work, as a major disruptor of circadian rhythm, may contribute to the development of metabolic dysfunction-associated steatotic liver disease (MASLD) by causing social jetlag (SJL), irregular eating patterns, and metabolic disturbances [5,6,7]. It has been reported that shift work, which disrupts the circadian rhythm, may also directly affect liver function. Working night shifts has been found to be positively associated with abnormal liver function tests in workers without MASLD [8]. In shift work, factors such as insufficient sleep duration or poor sleep quality, not just SJL or EJL, may also contribute to an increased risk of conditions such as obesity, metabolic syndrome, and MASLD [9,10].

MASLD was previously referred to as non-alcoholic fatty liver disease (NAFLD). Following the recent change in nomenclature, the term MASLD has replaced NAFLD. Previous studies have demonstrated that MASLD and NAFLD identify largely overlapping patient populations with comparable clinical characteristics and outcomes. Therefore, for the purposes of the present study, the terms were considered interchangeable where appropriate [11]. A systematic review and meta-analysis of population-based studies with survey years from 1990 to 2019 reported an overall global MASLD prevalence of approximately 30% [12]. In a forensic autopsy study of 60 adults in Türkiye, MASLD was identified in 43.3% of cases, and steatohepatitis was present in 46.1% of those with MASLD. However, the small and selected sample limits the generalizability of these findings [13]. In terms of management, resmetirom and semaglutide have received accelerated FDA approval in the United States for adults with noncirrhotic MASH and moderate-to-advanced fibrosis (F2–F3) [14,15]. Nevertheless, lifestyle modification remains a cornerstone of MASLD management [16].

Most previous studies conducted in Türkiye have focused primarily on the general population or clinical patients in hospital settings. Among shift workers, SJL, EJL, sleep duration and quality, and sedentary work patterns may constitute significant risk factors for the development of MASLD. Additionally, in a study conducted in Türkiye, although there was no significant association between chronotype and the severity of MASLD, it was found that insufficient sleep was significantly and positively associated with the severity of MASLD [17]. Anthropometric measurements reflecting body fat distribution [18,19,20] and adiposity indices [21,22] serve as practical indicators of this risk; however, no study has been identified that examines this risk in shift workers in conjunction with SJL and EJL.

Although circadian rhythm disruption and obesity-related factors have both been implicated in hepatic steatosis, their combined associations with liver fat accumulation among shift workers remain insufficiently understood. Therefore, the primary aim of this study was to examine the associations of shift-work status, SJL, EJL, sleep duration, and sleep quality with CAP, which is a surrogate marker for hepatic steatosis [23,24]. The secondary aim was to evaluate the sex-specific discriminatory performance of anthropometric measurements and adiposity indices for identifying hepatic steatosis, defined as CAP.

## 2. Materials and Methods

### 2.1. Study Design and Participants

This cross-sectional study was conducted at seven large-scale tea factories affiliated with ÇAYKUR, a state-owned enterprise in Rize, Türkiye. The required sample size was calculated as 322 using G*Power software (version 3.1.9.7) (type I error = 0.05, study power = 95%, and effect size = 0.48). The participating factories were visited on prearranged dates, and the study procedures were explained to employees in advance. On the scheduled data-collection day, employees who were willing to participate voluntarily attended the designated data-collection area. A total of 446 employees attended the data-collection session. Of these, eight were unable to complete the questionnaire and/or measurements because of work-related time constraints and had to leave before completion of the study procedures. In addition, body composition measurements could not be obtained from two participants because of technical limitations of the bioelectrical impedance analysis, including body size-related measurement limitations and failure to obtain a valid impedance reading. These 10 participants were excluded, resulting in a final study sample of 436 workers: 286 shift workers (256 men and 30 women) and 150 non-shift workers (72 men and 78 women), aged 19–64 years. Because participation was based on voluntary attendance, the number of employees who received information about the study but did not attend the data-collection session was not recorded.

Pregnant and breastfeeding women; individuals with chronic conditions such as mental or psychological disorders, eating disorders, diabetes, cardiovascular disease, or liver disease; those with a history of malignancy; and men and women consuming ≥20 g/day of alcohol were excluded from the study. Eligibility was assessed before enrolment by the occupational physician and the research team according to the predefined inclusion and exclusion criteria.

During the approximately four-month tea-processing season, shift workers followed a rotating three-shift schedule comprising 08:00–16:00, 16:00–00:00, and 00:00–08:00 shifts. Workers remained on the same shift for one week and rotated to the next shift the following week; therefore, each worker was assigned to the night shift once every three weeks. Workers had one rest day per week, which could be scheduled on any day according to worker preference and management approval and remained fixed during the tea-processing season. Among shift workers, length of employment represented the duration of occupational exposure to this seasonal rotating shift-work system, as they had worked under this schedule throughout their employment at the factory.

All data were collected through face-to-face interviews using a structured questionnaire. Information on participants’ sociodemographic and lifestyle characteristics, including age, sex, educational status, marital status, smoking status, and alcohol consumption, was collected during the interviews. Alcohol consumption was assessed during the face-to-face interview using interviewer-administered questionnaire items on the usual frequency of alcohol consumption, type of alcoholic beverage, and amount consumed. Sleep quality was assessed using the PSQI, while sleep- and meal-timing differences were assessed using SJL and EJL measures, respectively. Written informed consent was obtained from all participants before data collection. The study was conducted in accordance with the guidelines of the Declaration of Helsinki and was approved by the Recep Tayyip Erdoğan University Non-Interventional Clinical Research Ethics Committee (Project No: 2025/241).

### 2.2. Social Jetlag

SJL refers to the mismatch between social and biological rhythms and is commonly quantified from differences in sleep timing between workdays and free days, including sleep-corrected variants [3,25].

Sleep timing was assessed by self-report during face-to-face, interviewer-administered interviews. Participants reported their usual sleep time and wake-up time separately for workdays and free days. For shift workers, the free day referred to their weekly rest day, which was not necessarily a weekend day.

The sleep midpoint was calculated as the midpoint between the reported usual sleep time and wake-up time. SJL was calculated as the absolute difference between the sleep midpoint on free days and that on workdays. Higher SJL values indicated greater differences in sleep timing between workdays and free days. SJL was classified into three categories: normal (<1 h), moderate (1–<2 h), and severe (≥2 h) [26]. For rotating shift workers, workday sleep timing was assessed as self-reported usual timing rather than separately for morning, afternoon, and night shifts. The reported timing was not restricted to the shift assigned at the time of the interview. SJL values were expressed in absolute hours. During final data verification, one derived value stored with a negative sign was corrected to its absolute magnitude, consistent with this definition.

### 2.3. Eating Jetlag and Meal-Specific Jetlag

Eating jetlag (EJL) reflects differences in the timing of the daily eating window between workdays and free days. Meal timing was assessed by self-report during face-to-face, interviewer-administered interviews. All reported eating occasions were included, irrespective of whether food was brought from home, purchased, or provided by the employer.

Participants reported the timing of their first and last eating occasions separately for workdays and free days. They also reported their usual breakfast, lunch, and dinner times separately for workdays and free days. For rotating shift workers, meal timing was assessed as self-reported usual timing rather than separately for each shift type. The free day referred to their weekly rest day, which could occur on any day of the week depending on the work schedule.

The eating midpoint was calculated separately for workdays and free days as the midpoint between the first and last reported eating times, using the following formula: eating midpoint = first eating time + [(last eating time − first eating time)/2]. EJL was calculated as the absolute difference between the eating midpoints on workdays and free days. When the eating window extended beyond midnight, the interval between the first and last eating times was calculated according to the actual elapsed time across midnight, and the eating midpoint was then determined from this interval.

Meal-specific jetlag variables were calculated as the absolute differences in breakfast, lunch, and dinner times between workdays and free days. Because no validated cut-off value for EJL has been established, participants were categorized as having EJL or breakfast-, lunch-, or dinner-specific jetlag when the corresponding difference exceeded 1 h. Of note, a higher, data-driven threshold (≥3.5 h) has been reported for the association between EJL and body mass index [27].

Shift-work status was defined according to participants’ habitual work arrangement, distinguishing workers following the factory’s rotating three-shift schedule from those following a fixed non-shift schedule. The shift assigned at the time of the interview was not systematically collected or recorded. Participants reported their usual sleep and meal timing on workdays and free days, without separate assessments for each phase of the shift rotation cycle. Accordingly, SJL and EJL were calculated from these self-reported usual timing patterns and should not be interpreted as measurements specific to the interview-time shift or as verified averages across the entire rotation cycle.

### 2.4. Pittsburgh Sleep Quality Index (PSQI)

Sleep quality was assessed using the Pittsburgh Sleep Quality Index (PSQI). The PSQI is a self-assessment sleep quality questionnaire initially developed by Buysse et al. (1989) [28]. Ağargün (1996) conducted a Turkish version validity and reliability study [29]. A total score between 0 and 21 is obtained from the scale. A score between 0 and 5 shows good sleep quality, while a score of 6 and above shows poor sleep quality [29].

### 2.5. Assessment of Physical Activity Level

Physical activity was assessed using a 24-h physical activity record. Total energy expenditure was calculated by multiplying the time spent in each activity by the basal metabolic rate and the corresponding energy cost of the activity, expressed as the Physical Activity Ratio (PAR). Physical activity level (PAL) was then calculated by dividing total energy expenditure by basal metabolic rate. Participants were classified as lightly, moderately, or vigorously active according to the FAO/WHO/UNU classification criteria [30].

### 2.6. Anthropometric Measurements and Adiposity Indices

In this study, body weight, height, waist circumference (WC), hip circumference (HC), neck circumference and wrist circumference measurements were obtained as described previously [31]. Body mass index (BMI) was calculated as weight (kg) divided by height squared (m^2^). Waist-to-hip ratio (WHR) was calculated as WC divided by HC, and waist-to-height ratio (WHtR) was calculated as WC (cm) divided by height (cm). Overweight was defined as a BMI of 25.0–29.9 kg/m^2^, and obesity as a BMI of ≥30 kg/m^2^. Abdominal obesity was defined as WC ≥ 88 cm in females and ≥102 cm in males. An increase in metabolic risk is defined by a WHtR of ≥0.50. A WHR of >0.9 in men and >0.85 in women is defined as high metabolic risk [32,33,34]. For obesity and hepatic steatosis, the cutoff values for neck circumference were set at ≥40 cm for men and ≥34 cm for women [35].

The adiposity indices, including a body shape index (ABSI), body adiposity index (BAI), abdominal volume index (AVI), weight-adjusted waist index (WWI), conicity index (ConI), and hip index (HI), were calculated to analyse the distribution of fat in the body. Six adiposity indices were calculated following their standard measuring formulas [36,37,38].

-
$\mathrm{ABSI}={\mathrm{WC}}_{\left(m\right)}÷\left({\mathrm{BMI}}^{\frac{2}{3}}\times {\mathrm{Height}}_{\left(m\right)}^{\frac{1}{2}}\right)$

-
$\mathrm{BAI}={\mathrm{HC}}_{\left(\mathrm{cm}\right)}÷{\mathrm{Height}}_{\left(m\right)}^{1.5}-18$

-
$\mathrm{AVI}=\frac{2\times {\left({\mathrm{WC}}_{\left(\mathrm{cm}\right)}\right)}^{2}+0.7\times {\left({\mathrm{WC}}_{\left(\mathrm{cm}\right)}-{\mathrm{HC}}_{\left(\mathrm{cm}\right)}\right)}^{2}}{1000}$

-
$\mathrm{WWI}={\mathrm{WC}}_{\left(\mathrm{cm}\right)}÷{\mathrm{Weight}}_{\left(\mathrm{kg}\right)}^{\frac{1}{2}}$

-
$\mathrm{ConI}=\frac{{\mathrm{WC}}_{\left(m\right)}}{0.109\times \sqrt{\frac{{\mathrm{Weight}}_{\left(\mathrm{kg}\right)}}{{\mathrm{Height}}_{\left(m\right)}}}}$

-
${\mathrm{HI}}_{\mathrm{women}}={\mathrm{HC}}_{\left(\mathrm{cm}\right)}\times {{\mathrm{Height}}_{\left(\mathrm{cm}\right)}}^{0.310}\times {\mathrm{Weight}}_{\left(\mathrm{kg}\right)}^{-0.482}$

-
${\mathrm{HI}}_{\mathrm{men}}={\mathrm{HC}}_{\left(\mathrm{cm}\right)}\times {{\mathrm{Height}}_{\left(\mathrm{cm}\right)}}^{\frac{1}{5}}\times {\mathrm{Weight}}_{\left(\mathrm{kg}\right)}^{-\frac{2}{5}}$

### 2.7. Body Fat Analysis

In the analysis of body composition, an InBody 270 segmental bioelectrical impedance analyzer (InBody Co., Ltd., Seoul, Republic of Korea) was used to measure body fat mass and fat-free mass. BIA measurements were performed under standardized conditions in accordance with the manufacturer’s recommendations. Participants were measured barefoot and in a fasting state, without recent fluid intake; individuals who had recently performed strenuous exercise were not considered suitable for measurement. The fat-free mass index (FFMI) and fat mass index (FMI) were calculated as fat-free mass (kg)/height^2^ (m^2^) and body fat mass (kg)/height^2^ (m^2^), respectively. Body fat percentages of ≥25% in men and ≥32% in women were classified as excessive [39].

### 2.8. Hepatic Steatosis Screening

Hepatic steatosis was assessed using transient elastography with a portable FibroScan^®^ device (Echosens, Paris, France) by a certified specialist, with participants in the supine position. All transient elastography examinations were performed by a single highly experienced operator who had performed more than 10,000 examinations prior to and during the study period. The use of a single experienced operator minimized inter-operator variability and enhanced the consistency and reproducibility of the measurements. Hepatic steatosis was defined as a CAP value ≥ 248 dB/m, corresponding to at least mild hepatic steatosis (≥S1), based on the individual patient data meta-analysis by Karlas et al. [40] and the 2024 EASL–EASD–EASO Clinical Practice Guidelines [16].

Participants were instructed to fast and avoid caffeine for at least 3 h immediately before the examination, irrespective of their shift or sleep schedule. A valid measurement was available for 432 of the 436 participants; the remaining four participants were excluded from all CAP-based analyses. CAP and liver stiffness measurement (LSM) were used as surrogate markers of hepatic steatosis and fibrosis, respectively. All examinations were initially performed using an M probe. Based on real-time guidance from the automatic probe selection tool, which considers the skin-to-liver capsule distance, the probe was subsequently switched to XL when indicated. At least 10 valid measurements were obtained for each examination, and the median CAP and LSM values were recorded. For LSM, an interquartile range-to-median ratio (IQR/median) ≤ 0.30 was used as a reliability criterion. Examinations not meeting the predefined LSM reliability criteria were excluded from the analysis.

### 2.9. Statistical Analysis

All statistical analyses were performed using Jamovi (version 2.6.44) and IBM SPSS Statistics for Windows (version 22). The distribution of continuous variables was assessed for normality using the Shapiro–Wilk test and visual inspection of the distributions, separately within each sex-specific subgroup. Continuous variables in Tables 1 and 2 are presented as mean ± standard deviation (SD) for descriptive purposes, so the magnitude and dispersion of the anthropometric, body-composition and circadian rhythm-related measurements remain directly comparable with those reported in the occupational and MASLD literature; categorical variables are presented as frequency (*n*) and percentage (%). Because the distributions of most continuous variables did not meet the assumption of normality, the Mann–Whitney U test was used for the between-group comparisons between shift and non-shift workers; the test used for each variable is identified in the corresponding table footnote.

**Figure 1:**
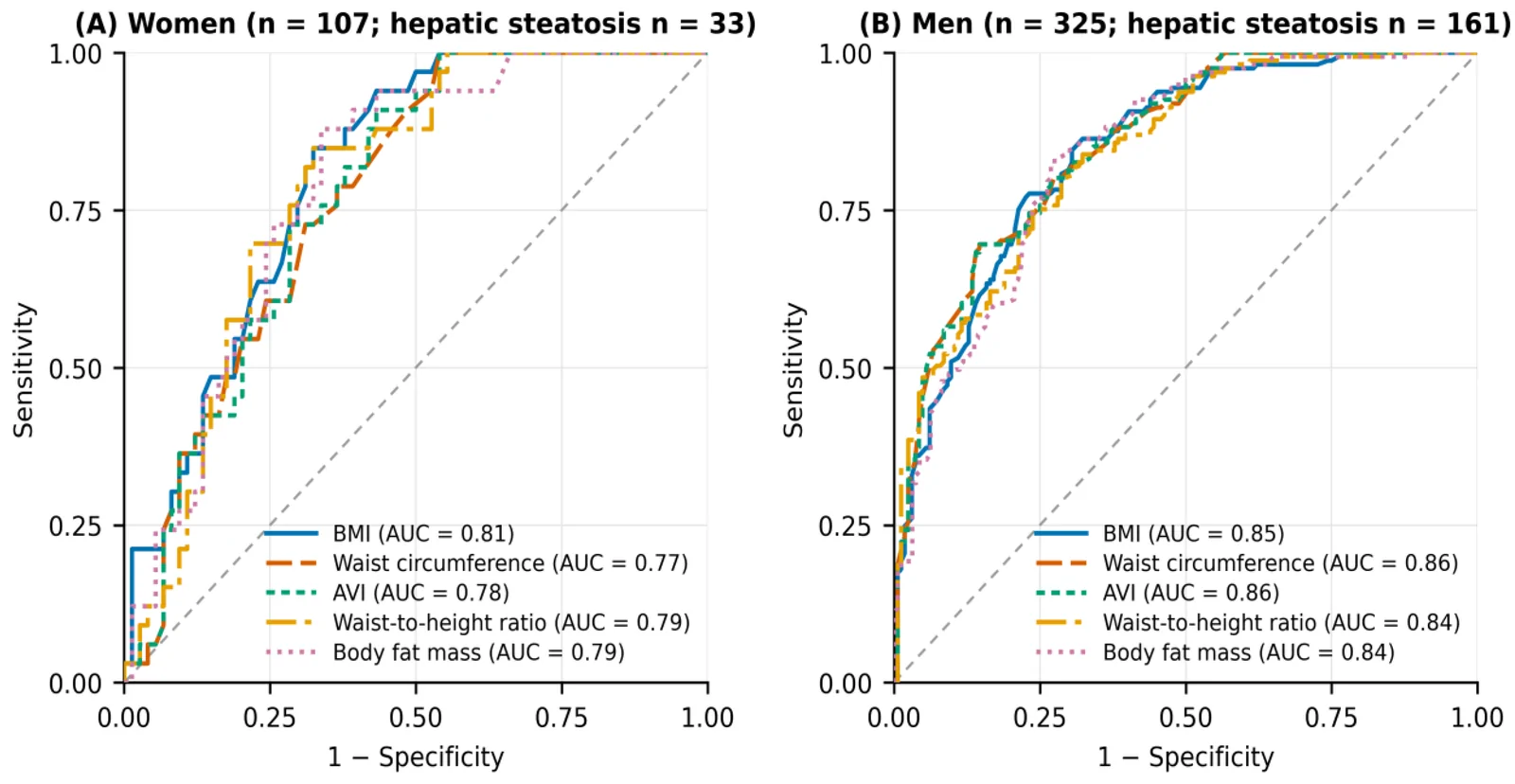
Sex-specific receiver operating characteristic curves of selected anthropometric and adiposity indicators for identifying hepatic steatosis defined as CAP ≥ 248 dB/m. (**A**) Women. (**B**) Men. The diagonal dashed line represents random classification (AUC = 0.50). Abbreviations: ROC, receiver operating characteristic; AUC, area under the receiver operating characteristic curve; AVI, abdominal volume index; BMI, body mass index.

Differences in categorical variables were assessed with the chi-square test. The descriptive summary and the inferential test address different questions and are therefore reported separately: the mean and SD describe the magnitude and dispersion of each measurement, whereas the reported *p*-values test whether the distributions differ between groups. Due to the non-normal distribution of the variables, Spearman’s rank correlation analysis was used to evaluate the relationships between SJL, EJL, sleep parameters, anthropometric measurements, body composition, and liver parameters. To account for potential confounding, rank-based partial Spearman correlation analyses were performed by rank-transforming the variables and correlating residuals after adjustment for the specified covariates using a hierarchical adjustment strategy. The crude model was unadjusted. Model 1 was adjusted for age and sex; Model 2 was additionally adjusted for shift-work status; Model 3 was further adjusted for physical activity level, smoking status, education level, marital status, and income status; and Model 4 was additionally adjusted for meal-skipping behavior. Correlation coefficients were reported as Spearman’s rank correlation coefficients (r_s_), together with raw *p*-values and BH-adjusted *q*-values. Multiple linear regression analyses were performed to identify factors independently associated with CAP. For the regression models, the unstandardized coefficient (B), standard error (SE), standardized coefficient (β), 95% confidence intervals (CIs), *t*-value, and *p*-value were reported. Additionally, model performance was evaluated using R, R^2^, adjusted R^2^, root mean square error (RMSE), and the overall F-statistic. The likelihood of multicollinearity was assessed using the variance inflation factor (VIF) values. Receiver operating characteristic (ROC) curve analysis was performed to evaluate the ability of anthropometric measurements, body composition parameters, adiposity indices, and circadian rhythm-related variables to identify hepatic steatosis. The area under the ROC curve (AUC), 95% CI, optimal cut-off values, sensitivity, and specificity were calculated. Optimal cut-off values were determined using Youden’s index. Because the optimal cut-off values were derived and evaluated in the same sample, without internal (e.g., bootstrap) or external validation, they may overestimate discriminatory performance and are presented as preliminary findings. ROC analyses were performed separately for men and women. Findings from the sex-specific analyses are presented in Table 5 and Figure 1. For the interpretation of AUC values, the following thresholds were applied: <0.60, poor; 0.60–0.70, acceptable; 0.70–0.80, fair; 0.80–0.90, good; and ≥0.90, excellent discriminatory performance. In all analyses, a *p*-value < 0.05 was considered statistically significant. Missing data were not imputed. Pairwise deletion was used in the correlation analyses, and listwise (complete-case) deletion was used in the regression and ROC analyses. Consequently, the number of participants differed between analyses; the fully adjusted regression model (Model A) included 381 participants, mainly because of missing values for EJL (*n* = 18), SJL (*n* = 13), smoking status (*n* = 8), body fat mass (*n* = 5), income status (*n* = 3), physical activity level (*n* = 3), and other covariates, whereas the simplified model (Model B), which did not include these variables, included the 432 participants with available CAP, age, BMI, and neck circumference data. Because the correlation and ROC analyses were exploratory and involved a large number of tests, a sensitivity analysis was performed in which the *p*-values were adjusted for multiple comparisons using the Benjamini–Hochberg false discovery rate (FDR) procedure, with the false discovery rate set at 5%. The correction was applied within each predefined family of tests, that is, within the 22 correlations of each model in Table 3 (five families) and within the 21 ROC analyses of each sex in Table 5 (two families). Tables 3 and 5 present both raw *p*-values and BH-adjusted *q*-values; the findings were interpreted with emphasis on the magnitude of the associations and their 95% CIs rather than on *p*-values alone.

## 3. Results

The general characteristics of the employees according to shift-work status are presented in Table 1. Among men, non-shift workers were older and had longer education and employment duration than shift workers (all *p* ≤ 0.023). PAL did not differ significantly by shift-work status in men (*p* = 0.086) or women (*p* = 0.980). Among women, shift workers had significantly greater mean SJL than non-shift workers (1.523 ± 2.179 vs. 0.843 ± 0.737 h, *p* = 0.017), and the distribution of SJL categories also differed significantly between the groups (*p* = 0.022). EJL and the prevalence of overall and meal-specific jetlag exceeding 1 h did not differ significantly by shift-work status in either sex. Although non-shift workers had numerically longer sleep duration and better sleep quality, these differences were not statistically significant.

The anthropometric measurements, body composition parameters, adiposity indices, and liver elastography findings of the employees are presented in Table 2. Among men, non-shift workers had lower body fat-free mass (60.3 ± 10.4 vs. 63.3 ± 14.5 kg, *p* = 0.004) and fat-free mass index (20.6 ± 2.0 vs. 21.4 ± 2.1 kg/m^2^, *p* = 0.004) than shift workers. Conversely, male non-shift workers had higher body fat percentage (25.4 ± 7.6% vs. 22.6 ± 7.5%, *p* = 0.007), FMI (7.5 ± 3.4 vs. 6.6 ± 3.2 kg/m^2^, *p* = 0.049), ABSI (0.084 ± 0.004 vs. 0.082 ± 0.006, *p* = 0.003), BAI (28.4 ± 3.9 vs. 27.1 ± 3.6, *p* = 0.012), ConI (1.33 ± 0.1 vs. 1.31 ± 0.1, *p* = 0.042), and WWI (11.0 ± 0.7 vs. 10.8 ± 0.9, *p* = 0.001). The prevalence of excess body fat was also higher among male non-shift workers than among male shift workers (20.6% vs. 11.0%, *p* = 0.038). None of the body composition or adiposity measures differed significantly between female shift and non-shift workers. CAP values and the prevalence of CAP-defined hepatic steatosis did not differ significantly by shift-work status in either sex. The proportion examined with the XL probe was similar in shift and non-shift workers in both sexes (men: 9.4% vs. 8.3%, *p* = 0.863; women: 6.7% vs. 12.8%, *p* = 0.352).

Table 3 presents the crude and adjusted partial correlations between CAP and anthropometric, adiposity, circadian rhythm, and sleep-related variables. In crude analyses, CAP was positively correlated with all anthropometric and adiposity-related indices. Moderate correlations were observed for WC (*r_s_* = 0.68, *p* < 0.001), AVI (*r_s_* = 0.68, *p* < 0.001), BMI (*r_s_* = 0.67, *p* < 0.001), and WHtR (*r_s_* = 0.66, *p* < 0.001). Moderate positive correlations were also found for NC, body fat mass, FFMI, FMI, ConI, HI, and WWI (all *p* < 0.001). After adjustment for age and sex in Model 1 and the additional adjustment for shift-work status in Model 2, the overall pattern of correlations remained consistent. Model 3 was further adjusted for physical activity level, smoking status, education level, marital status, and income status, whereas Model 4 additionally included meal-skipping status. In Model 4, WC, AVI, BMI, and body fat mass showed the strongest correlations with CAP (all *r_s_* = 0.66), followed by WHtR (*r_s_* = 0.65) and FMI (*r_s_* = 0.63; all *p* < 0.001). Among the anthropometric and adiposity variables, ABSI showed the weakest but still statistically significant correlation with CAP in Model 4 (*r_s_* = 0.14, raw *p* = 0.008, *q* = 0.011). None of the circadian rhythm-related variables, including SJL, EJL, breakfast jetlag, lunch jetlag, and dinner jetlag, were significantly correlated with CAP in either the crude analysis or any of the adjusted models. Similarly, sleep duration and PSQI total score were not significantly correlated with CAP in any model (all *p* > 0.05). After Benjamini–Hochberg correction within each model, all correlations between CAP and the anthropometric and adiposity-related variables remained statistically significant in every model (all adjusted *q* ≤ 0.023, with the largest adjusted *q*-value observed for ABSI), whereas none of the circadian rhythm- or sleep-related variables reached statistical significance in any model (all adjusted *q* ≥ 0.160).

The results of the multivariable linear regression analyses are presented in Table 4. In the fully adjusted model (Model A), which included 381 participants with complete data, age, BMI, and neck circumference were independently associated with higher CAP values. The model explained 49% of the variance in CAP (R^2^ = 0.49; adjusted R^2^ = 0.45; overall model *p* < 0.001). Age (β = 0.12, *p* = 0.014), BMI (β = 0.31, *p* = 0.023), and neck circumference (β = 0.29, *p* < 0.001) were statistically significant predictors. Each 1-cm increase in neck circumference was associated with a 3.98-dB/m higher CAP value (95% CI: 2.12 to 5.85), each 1-kg/m^2^ increase in BMI was associated with a 3.21-dB/m higher CAP value (95% CI: 0.44 to 5.98), and each 1-year increase in age was associated with a 0.65-dB/m higher CAP value (95% CI: 0.13 to 1.17). Waist circumference, body fat mass, fat-free mass, SJL, EJL, sleep duration, and PSQI total score were not statistically significantly associated with CAP after adjustment (all *p* ≥ 0.142). Multicollinearity was not considered problematic, as VIF values ranged from 1.11 to 3.60.

A simplified model (Model B), including age, BMI, and neck circumference, was examined in 432 participants with complete data for these variables. The model explained 45% of the variance in CAP (R^2^ = 0.45; adjusted R^2^ = 0.44; overall model *p* < 0.001). All three variables remained independently associated with higher CAP values: age (β = 0.10, *p* = 0.006), BMI (β = 0.40, *p* < 0.001), and neck circumference (β = 0.30, *p* < 0.001). Because Models A and B were estimated using different numbers of participants, their model-fit statistics should not be directly compared.

The sex-specific discriminatory performance of anthropometric measurements, body composition parameters, adiposity indices, and circadian rhythm- and sleep-related variables for identifying hepatic steatosis, defined as CAP ≥248 dB/m, is presented in Table 5 and Figure 1. In men, the highest AUC values were observed for WC and AVI (both AUC = 0.86, 95% CI: 0.82–0.90), followed by BMI (AUC = 0.85, 95% CI: 0.80–0.89), body fat mass and WHtR (both AUC = 0.84), body weight and FMI (both AUC = 0.83), and neck circumference and WHR (both AUC = 0.82; all *p* < 0.001). At a cut-off of 27.75 kg/m^2^, BMI showed 77.6% sensitivity and 76.8% specificity, whereas at a cut-off of 102.2 cm, WC showed 69.6% sensitivity and 85.4% specificity. In women, BMI had the highest AUC (0.81, 95% CI: 0.73–0.89), followed by body fat mass, FMI, and WHtR (all AUC = 0.79) and body weight and AVI (both AUC = 0.78; all *p* < 0.001). At a cut-off of 27.0 kg/m^2^, BMI showed 84.9% sensitivity and 67.6% specificity, whereas at a cut-off of 84.5 cm, WC showed 100.0% sensitivity and 46.0% specificity. ABSI did not significantly discriminate hepatic steatosis in women (AUC = 0.46, raw *p* = 0.500, *q* = 0.525) and showed limited discriminatory performance in men (AUC = 0.62, raw *p* < 0.001, *q* < 0.001). Social jetlag and EJL did not significantly discriminate hepatic steatosis in either sex. Sleep duration and PSQI total score were also not significant in men. In women, sleep duration showed weak discrimination (AUC = 0.63, 95% CI: 0.52–0.74; raw *p* = 0.030, *q* = 0.042), whereas PSQI total score was not significant (AUC = 0.61, 95% CI: 0.50–0.73; raw *p* = 0.057, *q* = 0.071).

## 4. Discussion

This study aimed to examine the associations of SJL, EJL, sleep duration, sleep quality, anthropometric measurements, and adiposity indices with CAP among shift and non-shift workers and to evaluate their independent associations and discriminatory performance using multivariable regression and receiver operating characteristic analyses. While the male and female ratio is similar among non-shift workers, the majority of shift workers are male. Although labor force participation rates may vary by gender, the concentration of men in sectors where production, industry, and night work are prevalent increases their proportion among shift workers. While women primarily work in service and office jobs suited to regular daytime shifts, it has been reported that workers of different ethnic backgrounds are more likely to work both day and night shifts [41].

In the present study, the distribution of SJL categories differed significantly between female shift and non-shift workers (*p* = 0.022), with moderate (1 to <2 h) and severe (≥2 h) SJL being more frequent among shift workers. Although moderate and severe SJL were also more prevalent among male shift workers, the difference was not statistically significant (*p* = 0.542). The different SJL category distributions among female shift workers coincided with a higher frequency of meal skipping; however, this finding should not be interpreted as evidence that SJL caused meal skipping. Moreover, possible explanations involving time constraints, concerns about body weight, or family responsibilities were not directly assessed and therefore remain speculative. ÇAYKUR provides scheduled lunch, dinner, and night-shift meals for shift workers. This workplace meal provision may have contributed to the relatively low frequency of meal skipping and the limited variation in meal timing, although its effect was not directly evaluated. Mean EJL was below 1 h in all groups. The proportion of workers with EJL exceeding 1 h ranged from 28.1% to 39.7% and did not differ significantly by shift-work status in either men or women. Across both sexes and shift-work groups, the proportion of participants with meal-specific jetlag exceeding 1 h was highest for breakfast and lowest for dinner (Table 1). This pattern may partly reflect differences in the flexibility of meal schedules throughout the day. However, because meal timing was not assessed separately for different shift types, the potential contribution of rotating shift schedules to these differences could not be determined.

Among women, both mean SJL (*p* = 0.017) and the distribution of SJL categories (*p* = 0.022) differed significantly between shift and non-shift workers. However, neither sleep duration nor PSQI total score differed significantly by shift-work status among women (Table 1). These separate group comparisons do not establish whether SJL is directly associated with sleep duration or sleep quality. The relatively small number of female shift workers (*n* = 30) and the limited comparability of SJL estimates between rotating shift and non-shift workers warrant cautious interpretation of these findings. Previous studies have indicated that night and rotating shift work may adversely affect sleep duration and sleep quality, although findings may differ according to sex and work-schedule characteristics [2,42]. Lecca et al. reported greater daytime sleepiness and poorer sleep quality among women, suggesting that sex-specific factors may influence adaptation to night-shift work [42]. In addition, the association between SJL and sleep deficit makes it difficult to distinguish the effects of circadian misalignment from those of sleep loss [2]. In the present study, the proportion of participants reporting 7–8 h of sleep per day was broadly similar across shift-work groups in both sexes. Although sleep duration of <7 h/day was numerically more common among shift workers than among non-shift workers, the difference was not statistically significant. This finding differs from recent large-scale evidence showing that shift work is associated with higher odds of sleep disturbance, with the greatest odds observed among individuals who consistently work night shifts [43]. Evidence regarding sleep duration and hepatic steatosis remains inconsistent. A meta-analysis of 15 studies found that short sleep duration was associated with a modestly higher risk of MASLD, although substantial between-study heterogeneity was present [10]. In the present study, however, neither sleep duration nor PSQI total score was significantly associated with CAP in the crude or adjusted analyses. These findings suggest that average sleep duration and self-reported sleep quality, when considered individually, may not fully capture the multidimensional aspects of sleep and circadian disruption potentially relevant to hepatic steatosis. Beyond average sleep duration and quality, the timing and regularity of sleep, food intake, and physical activity may also influence behavioral alignment with the circadian system. Differences in study populations, sleep definitions, working-hour patterns, shift schedules, and methods used to assess hepatic steatosis may partly account for the inconsistent findings. Park and Lee reported that, among individuals sleeping 5–6 h per day, working more than 52 h per week was associated with higher odds of MASLD compared with working 40–52 h per week, whereas no significant association was observed among those sleeping ≤5 or ≥7 h per day [44]. In a large cohort of young workers, shift work was associated with incident MASLD among workers in their twenties. The association remained statistically significant in stratified analyses among women and participants with poor sleep quality; however, the corresponding interaction tests were not significant [9]. Conversely, Liu et al. found that long nighttime sleep duration, rather than short sleep duration, was associated with incident MASLD among middle-aged and older adults [45]. Sleep regularity may also provide information beyond average sleep duration, as both short sleep duration and greater sleep irregularity have been associated with higher odds of MASLD [46]. In the present study, PSQI-defined sleep quality did not differ significantly between shift and non-shift workers in either sex. Although the regular three-shift rotation may partly explain this finding, its effect was not directly assessed.

In a four-year prospective cohort of Chinese rail workers, the cumulative incidence of MASLD was 30.43%, and frequent shift work was associated with a modestly higher incidence of MASLD [47]. A recent systematic review of nine observational studies found that most reported positive associations between shift work and MASLD, while imprecise exposure and outcome measurements limited causal interpretation [48]. In the present study, neither the prevalence of CAP-defined hepatic steatosis nor the distribution of CAP values across quartiles differed significantly between shift and non-shift workers, although hepatic steatosis was more prevalent among men than women. Tang et al. reported a hepatic steatosis prevalence of 45.5% among workers, with a higher prevalence and a less favorable lifestyle and metabolic risk profile among men than women [49]. Zhang et al. found no significant association between the evaluated night-shift exposure metrics and MASLD among female workers. Among male steelworkers, however, current night-shift work, longer duration, greater cumulative exposure, higher monthly frequency, and longer average night shifts were associated with higher odds of MASLD [50]. Large cross-sectional studies have also reported associations of shift work, long working hours, and heavy manual work with elevated non-invasive hepatic steatosis risk scores; however, these studies relied on surrogate indices rather than imaging-based diagnoses and cannot establish causality [51,52]. Overall, the available evidence suggests a possible association between shift-related occupational exposures and hepatic steatosis, but the consistency and magnitude of this association remain uncertain because of substantial heterogeneity in study design, exposure assessment, and outcome definition. Transient-elastography screening of apparently healthy airline pilots has likewise shown that clinically silent steatosis and fibrosis can be detected in occupational cohorts [53]. An occupational study of university staff also examined associations of metabolic and occupational factors with fatty liver severity [54].

The World Health Organization defines overweight and obesity as abnormal or excessive fat accumulation that poses a health risk [34]. A study of healthcare workers reported higher body weight, BMI, and waist circumference among night-shift workers compared with day-shift workers, with the association between night-shift work and abdominal obesity remaining significant after adjustment for age and sex [55]. In the present study, however, neither BMI nor BMI category distribution differed significantly by shift-work status in either sex, although the prevalence of overweight and obesity was high in both groups, broadly consistent with national estimates for Türkiye reported in the WHO European Regional Obesity Report 2022 [34]. Obesity, particularly abdominal obesity, is a major metabolic risk factor associated with the development and progression of MASLD [16]. In the present study, BMI was strongly and positively correlated with CAP values in both crude and adjusted analyses, and the magnitude of this association remained largely unchanged after adjustment for demographic, occupational, lifestyle, and meal-related factors. Body fat mass, FMI, FFMI, and several adiposity indices were also positively correlated with CAP values, with generally similar patterns across the adjusted models. Although BMI is widely used to classify obesity in adults, it does not distinguish fat mass from fat-free mass or indicate body fat distribution [56]. Bioelectrical impedance analysis is commonly used to estimate body composition, whereas circumferential measurements such as WC, hip circumference, and neck circumference provide indirect indicators of central or overall adiposity [57]. Among male shift workers, BIA-derived FFM and height-adjusted FFMI were significantly higher than in non-shift workers, whereas body fat percentage, FMI, and several adiposity indices, including ABSI, BAI, ConI, and WWI, were significantly lower (Table 2). These differences may reflect variation in occupational physical demands, habitual activity patterns, or other unmeasured factors; however, occupational activity intensity was not directly quantified, and causal inferences cannot be made. Previous research in male steelworkers has shown that BIA-derived body fat mass and FMI may help discriminate severe steatosis [58]. FMI, which standardizes fat mass for height, has also been associated with hepatic steatosis and fibrosis and may provide information beyond BMI alone [59,60].

Simple anthropometric measures, including waist circumference, waist-to-hip ratio, and waist-to-height ratio, have been reported to provide useful indicators of visceral adiposity and to aid in identifying individuals at higher risk of MASLD [61]. Waist circumference has therefore been recommended for assessment even among individuals without an elevated BMI [62]. Body mass index, WC, WHtR, and subcutaneous fat have also shown good discriminatory performance for MASLD in some populations while offering practical advantages such as non-invasiveness, ease of use, and low cost [63]. Consistent with previous evidence, waist circumference, neck circumference, and waist-to-height ratio remained strongly correlated with CAP values across the adjusted models. The magnitude of these associations changed only minimally after sequential adjustment for shift-work status, lifestyle characteristics, and meal-skipping behavior, indicating that these measures were consistent correlates of CAP-defined hepatic steatosis in this sample.

Anthropometric measurements can be used to calculate adiposity indices that provide indirect information on body size and fat distribution [64,65]. In the present study, all adiposity indices, including ABSI, were positively associated with CAP values. Among these indices, AVI showed the strongest correlation among the derived indices, followed by FMI, waist-to-hip ratio, HI, and ConI; ABSI showed the weakest positive association. In a similar study, BMI in men and WHtR in women were reported as the indices with the highest potential for predicting MASLD, and, similarly to our study, ABSI was found to be the least effective index in both genders [66]. It was reported that WWI could be used as a simple anthropometric index to predict hepatic steatosis [67]. Overall, anthropometric measurements and derived adiposity indices may provide practical adjunctive tools for assessing hepatic steatosis risk, although they should not be considered substitutes for imaging-based assessment [68].

The present study showed that anthropometric and adiposity-related measures were more strongly associated with CAP values than the circadian rhythm and sleep-related variables examined. In the correlation analyses, SJL, EJL, meal-timing jetlag, sleep duration, and PSQI total score were not significantly associated with CAP values in either the crude or adjusted models, whereas most anthropometric and adiposity measures showed moderate-to-strong positive correlations. Previous studies have reported associations of shift work and circadian syndrome with MASLD risk [51,69]; however, comparable associations with the circadian and sleep-related variables assessed in the present study were not observed. Consistent with the correlation findings, age, BMI, and neck circumference remained independently associated with CAP values in the fully adjusted multivariable model, whereas social jetlag, eating jetlag, sleep duration, and PSQI total score remained nonsignificant. BMI and neck circumference showed the largest standardized coefficients among the anthropometric variables, indicating that overall adiposity and the body-fat distribution indirectly reflected by neck circumference were more closely associated with hepatic steatosis in this sample. Experimental evidence suggests that social jetlag can perturb endocrine circadian rhythms and hepatic lipid metabolism [70], but this mechanism cannot be inferred from the present cross-sectional human data.

In contrast, social jetlag, eating jetlag, sleep duration, PSQI total score, waist circumference, body fat mass, and fat-free mass were not independently associated with CAP values in the fully adjusted model. These findings indicate that the circadian and sleep-related variables assessed in this study were not independently associated with CAP values after adjustment for the other variables included in the model. However, because formal mediation analysis was not performed, these findings do not establish that adiposity mediates a potential relationship between circadian disruption and hepatic steatosis. Neck circumference has been proposed as a practical indirect marker of upper-body adiposity and has been associated with cardiometabolic abnormalities and hepatic steatosis in previous studies, whereas BMI remains a widely used, although nonspecific, indicator of overall adiposity in clinical and epidemiological research [71,72,73,74].

The ROC analyses showed that anthropometric and adiposity-related measures generally had higher observed discriminatory performance for CAP-defined hepatic steatosis than the circadian rhythm- and sleep-related variables, particularly among men. These findings are consistent with evidence indicating that visceral adiposity is more closely associated with hepatic fat accumulation and MASLD risk than general adiposity measures alone [16]. Previous studies have reported that anthropometric and adiposity-related indices may provide useful discriminatory information for hepatic steatosis and MASLD, although their performance varies across populations and according to the specific index evaluated [61,63,64,68]. In men, WC and AVI showed good discriminatory performance (both AUC = 0.86), while BMI showed a comparable observed AUC of 0.85. In women, BMI had the highest observed AUC and showed good discriminatory performance (AUC = 0.81), whereas body fat mass and FMI showed fair performance (both AUC = 0.79). BAI showed similar fair discriminatory performance in women and men, with AUCs of 0.77 and 0.75, respectively. In contrast, ABSI showed no significant discriminatory ability in women (AUC = 0.46, raw *p* = 0.500, *q* = 0.525) and only limited discrimination in men (AUC = 0.62, raw *p* < 0.001, *q* < 0.001). The limited discriminatory performance of ABSI is consistent with previous studies reporting weak performance for this index in both sexes [66,75]. Although the observed sex-stratified patterns may partly reflect differences in body composition and fat distribution, the AUCs were not formally compared between sexes and should therefore be interpreted cautiously. Larger sex-stratified studies are needed to confirm these findings.

The optimal cut-off values identified in the present study provide preliminary information on the discriminatory performance of anthropometric measures for CAP-defined hepatic steatosis. In women, the optimal WC cut-off was 84.5 cm, which was below the WHO high-risk threshold of 88 cm and yielded a sensitivity of 100.0% but a specificity of only 46.0%. Thus, a lower threshold may improve sensitivity in this occupational population, although the associated low specificity would result in a substantial number of false-positive classifications. In men, the optimal BMI cut-off was 27.75 kg/m^2^, within the overweight rather than obese range, suggesting that CAP-defined hepatic steatosis may already be present at BMI levels below the conventional obesity threshold. These findings are consistent with evidence that optimal anthropometric cut-offs for MASLD may vary across populations and may be lower than conventional obesity thresholds in some Asian cohorts [75]. In contrast, SJL, EJL, sleep duration, and PSQI total score showed little or no discriminatory ability for CAP-defined hepatic steatosis in men, with AUC values close to 0.50 and no statistically significant results. In women, sleep duration was statistically significant and remained so after Benjamini–Hochberg correction (AUC = 0.63; raw *p* = 0.030; *q* = 0.042), whereas PSQI total score was not (AUC = 0.61; raw *p* = 0.057; *q* = 0.071); both AUCs indicated only weak discrimination, with relatively wide confidence intervals. Because the AUCs were not formally compared between sexes and sleep was assessed using self-reported measures, the apparent sex-stratified pattern should be interpreted cautiously and confirmed using larger samples and objective sleep measures. Collectively, the findings indicate that simple anthropometric measures, particularly those reflecting abdominal adiposity, may provide practical and low-cost information for the initial identification of individuals more likely to have hepatic steatosis. However, these measures should be considered adjunctive rather than diagnostic and require validation in other populations [16,68].

### Strengths and Limitations

A major strength of this study is the simultaneous evaluation of shift-work status, circadian rhythm and sleep-related characteristics, anthropometric measurements, adiposity indices, body composition parameters, and CAP-defined hepatic steatosis in an occupational population recruited from seven tea factories. This comprehensive approach allowed the relative associations of circadian, sleep-related, and adiposity-related factors with hepatic steatosis to be examined within the same study framework. Despite its strengths, this study has several important limitations. Because of the cross-sectional design, causal or temporal relationships cannot be established. A major methodological limitation concerns the assessment of SJL and EJL among rotating shift workers. The primary comparison involved workers following a weekly rotating shift schedule and those working under a fixed non-shift schedule, rather than comparisons between individual shift types. Participants were asked to report their usual sleep and meal timing on workdays and free days, without separate assessments for morning, afternoon, and night shifts. Furthermore, shift assignments at the time of the interview were not systematically recorded, preventing the reporting of a reliable distribution by interview shift and the performance of shift-phase-specific sensitivity analyses. Although the reported timings were intended to reflect usual patterns, they may not adequately capture within-person variations across the rotation cycle and may have been influenced by the particular shift worked during the assessment period. Consequently, SJL and EJL estimates cannot be considered directly comparable among shift workers interviewed during different shift phases or between shift and non-shift workers. This limitation directly affects the shift-work comparison component of the study’s main aim and restricts the interpretation of the observed SJL differences, particularly among women, which should be considered exploratory rather than definitive. Additionally, the PSQI assesses general sleep quality rather than shift-specific sleep disruption, and biochemical parameters and detailed food-consumption records were unavailable.

The single-region occupational sample and voluntary participation may limit the generalizability of the findings and introduce selection bias. Furthermore, the small number of female shift workers (*n* = 30) may have reduced the precision of comparisons by shift-work status among women, while the smaller female sample overall warrants caution when interpreting sex-specific ROC estimates. The ROC-derived cut-off values were selected and evaluated within the same study population, potentially leading to an overestimation of discriminatory performance. Therefore, these cut-offs should be considered preliminary and require external validation in independent, sex-stratified cohorts before broader application. Although the Benjamini–Hochberg procedure was applied to the correlation and ROC analyses, the large number of exploratory comparisons warrants cautious interpretation, particularly for findings close to the statistical significance threshold.

Finally, hepatic steatosis was assessed using CAP rather than a formal diagnosis of MASLD. Although CAP is an established non-invasive indicator of hepatic steatosis, it does not capture the full diagnostic spectrum of MASLD. Nevertheless, given the high prevalence of MASLD and the central role of hepatic steatosis in its definition, our findings may also be relevant to populations with MASLD, although their applicability to this population remains uncertain.

Future longitudinal, multicentre studies incorporating detailed assessments of shift types and rotation patterns, repeated measurements of sleep and meal timing across different phases of the rotation cycle, comprehensive dietary and biochemical evaluations, and independent sex-stratified validation cohorts are needed to clarify temporal relationships and assess the generalizability and clinical applicability of these findings.

## 5. Conclusions

This study comprehensively evaluated circadian rhythm and sleep-related characteristics, anthropometric measurements, body composition parameters, and adiposity indices in relation to CAP-defined hepatic steatosis among shift and non-shift workers. Among women, both mean SJL (*p* = 0.017) and the distribution of SJL categories (*p* = 0.022) differed significantly by shift-work status, whereas these differences were not statistically significant among men. CAP values and the prevalence of CAP-defined hepatic steatosis did not differ significantly by shift-work status in either sex. SJL, EJL, sleep duration, and PSQI total score were not significantly associated with CAP values after adjustment for potential confounders. In contrast, anthropometric and adiposity-related measures showed stronger and more consistent associations with CAP values. In the fully adjusted model, age, BMI, and neck circumference remained independently associated with higher CAP values. Sex-stratified ROC analyses showed that WC and AVI had the highest observed AUCs in men, whereas BMI had the highest observed AUC in women. SJL and EJL did not significantly discriminate hepatic steatosis in either sex, while sleep duration showed weak discriminatory performance in women. However, because shift-specific sleep and meal timing were not assessed, SJL and EJL estimates cannot be considered directly comparable between shift and non-shift workers. Therefore, the observed SJL differences among women should be interpreted as exploratory findings, limiting definitive conclusions regarding shift-work-related differences.

The findings regarding anthropometric and adiposity-related measures suggest that measurements reflecting overall and central adiposity may be particularly informative in relation to CAP-defined hepatic steatosis. However, these measures should be regarded as adjunctive tools for prioritizing individuals for further evaluation rather than as substitutes for established assessment methods, such as CAP measurement using FibroScan. Given the cross-sectional design and the evaluation of ROC-derived thresholds within the same sample, causal interpretations and immediate clinical application of the proposed cut-offs are not warranted. These simple, readily obtainable anthropometric measurements and derived adiposity indices may facilitate preliminary risk stratification for hepatic steatosis in occupational settings.

## Figures and Tables

**Table 1:** General characteristics of employees according to shift-work status.

Variables	Shift Work (*n*: 286)		Non-Shift Work (*n*: 150)		*p*1 Value	*p*2 Value
	Male (256)	Female (30)	Male (72)	Female (78)		
	*n* (%)	*n* (%)	*n* (%)	*n* (%)		
**Age, (years) (mean ± SD)**	37.5 ± 9.7	38.0 ± 9.0	41.2 ± 10.0	40.6 ± 8.8	**0.018**	0.256
**Total duration of education, year (Mean ± SD)**	11.2 ± 4.0	15.1 ± 1.7	12.8 ± 4.0	14.9 ± 2.9	**0.001**	0.703
**Length of employment, year (Mean ± SD)** (Male *n*: 269; Female *n*: 100)	8.3 ± 8.0	12.4 ± 8.1	12.3 ± 11.0	13.6 ± 10.5	**0.023**	0.912
**Marital status** (Male *n*: 327; Female *n*: 108)						
Married	147 (57.4)	23 (76.7)	46 (64.8)	55 (70.5)	0.279	0.634
Single	109 (42.6)	7 (23.3)	25 (35.2)	23 (29.5)		
**Number of main meals (mean ± SD)** (Male *n*: 325; Female *n*: 105)	2.5 ± 0.6	2.3 ± 0.5	2.6 ± 0.5	2.3 ± 0.6	0.173	1.000
**Number of snacks (mean ± SD)** (Male *n*: 255; Female *n*: 86)	1.2 ± 0.9	1.4 ± 0.6	1.1 ± 1.0	1.3 ± 0.8	0.491	0.544
**Meal skipping status** (Male *n*: 327; Female *n*: 108)						
Yes/sometimes	200 (78.4)	27 (90.0)	48 (66.7)	64 (83.1)	0.113	**0.030**
No	55 (21.6)	3 (10.0)	24 (33.3)	13 (16.9)		
**Social jetlag, h, (mean ± SD)** (Male *n*: 316; Female *n*: 107)	0.673 ± 0.953	1.523 ± 2.179	0.666 ± 1.028	0.843 ± 0.737	0.827	**0.017**
Normal (<1 h)	162 (65.6)	11 (36.7)	49 (71.0)	46 (59.7)	0.542	**0.022**
Moderate (1-<2 h)	61 (24.7)	13 (43.3)	16 (23.2)	27 (35.1)		
Severe (≥2 h)	24 (9.7)	6 (20.0)	4 (5.8)	4 (5.2)		
**Eating jetlag, h (mean ± SD)** (Male *n*: 313; Female *n*: 105)	0.58 ± 1.1	0.64 ± 0.6	0.65 ± 0.8	0.79 ± 0.7	0.185	0.317
Eating jetlag (>1 h)	72 (28.1)	11 (36.7)	24 (33.3)	31 (39.7)	0.384	0.829
Breakfast jetlag (>1 h)	103 (40.2)	17 (56.7)	33 (45.8)	46 (59.0)	0.418	0.831
Lunch jetlag (>1 h)	66 (25.8)	8 (26.7)	20 (27.8)	23 (29.5)	0.762	0.817
Dinner jetlag (>1 h)	42 (16.4)	7 (23.3)	15 (20.8)	20 (25.6)	0.382	1.000
**Sleep duration, h (mean ± SD)** (Male *n*: 326; Female *n*: 108)	7.0 ± 1.3	7.0 ± 1.5	7.2 ± 1.4	7.4 ± 1.3	0.341	0.314
Short sleep (<7 h)	79 (31.0)	12 (40.0)	16 (22.2)	18 (23.4)	0.095	0.184
Healthy sleep (7–8 h)	163 (63.9)	14 (46.7)	48 (66.7)	50 (64.9)		
Long sleep (≥9 h)	13 (5.1)	4 (13.3)	8 (11.1)	9 (11.7)		
**PSQI (m** **ean ± SD)**	4.7 ± 2.9	5.2 ± 2.4	4.5 ± 2.5	4.6 ± 2.4	0.564	0.276
Good sleep quality (≤5)	177 (69.1)	19 (63.3)	51 (70.8)	55 (70.5)	0.885	0.495
Poor sleep quality (≥6)	79 (30.9)	11 (36.7)	21 (29.2)	23 (29.5)		
**Smoking status** (Male *n*: 324; Female *n*: 104)						
Yes	156 (61.7)	10 (35.7)	36 (50.7)	19 (25.0)	0.140	0.131
No	97 (38.3)	18 (64.3)	35 (49.3)	57 (75.0)		
**Physical activity level (PAL) (mean ± SD)**	1.97 ± 0.33	1.96 ± 0.43	1.90 ± 0.29	1.91 ± 0.32	0.086	0.980

Chi-square (χ2) test used for nominal data, *n* (%). Continuous variables are presented as mean ± SD and were compared using two-sided Mann–Whitney U tests. A two-sided *p* <0.05 was considered statistically significant and is indicated in bold. *p*1 value: difference between shift work and non-shift work in men, *p*2 value: difference between shift work and non-shift work in women; sample sizes vary across variables because of missing data.

**Table 2:** Anthropometric measurements, body composition, adiposity indices, and CAP- and LSM-based liver parameters according to shift-work status.

	Shift Work (*n* = 286)		Non-Shift Work (*n* = 150)		*p*1 Value	*p*2 Value
	Male (256)	Female (30)	Male (72)	Female (78)		
	Mean ± SD	Mean ± SD	Mean ± SD	Mean ± SD		
**Body mass index, kg/m^2^**	28.1 ± 5.0	26.9 ± 5.5	27.9 ± 4.6	27.6 ± 5.2	0.919	0.524
18.5–24.9 (normal) (*n*, %)	71 (27.7)	12 (40.0)	19 (26.4)	29 (37.2)	0.865	0.914
25–29.9 (overweight) (*n*, %)	106 (41.4)	10 (33.3)	33 (45.8)	25 (32.1)		
≥30 (obese) (*n*, %)	79 (30.9)	8 (26.7)	20 (27.8)	24 (30.8)		
**Waist circumference, cm**	100.5 ± 14.5	87.6 ± 14.3	100.7 ± 12.6	91.0 ± 12.5	0.797	0.25
Male ≥ 102 cm; Female ≥ 88 cm (*n*, %)	110 (43.0)	14 (46.7)	32 (44.4)	40 (51.3)	0.932	0.173
**Neck circumference, cm**	40.3 ± 3.3	35.2 ± 3.4	40.4 ± 3.0	36.2 ± 3.6	0.693	0.215
Male, ≥40 cm; Female ≥ 34 cm (*n*, %)	141 (55.1)	20 (66.7)	40 (55.6)	56 (71.8)	0.943	0.601
**Waist-to-hip ratio**	0.95 ± 0.1	0.83 ± 0.1	0.96 ± 0.1	0.85 ± 0.1	0.391	0.438
Male > 0.90; Female > 0.85 (*n*, %)	198 (77.3)	13 (43.3)	61 (84.7)	36 (46.2)	0.175	0.792
**Waist-to-height ratio**	0.58 ± 0.08	0.55 ± 0.09	0.58 ± 0.07	0.56 ± 0.08	0.216	0.335
Male-Female ≥ 0.5 (*n*, %)	211 (82.4)	19 (63.3)	66 (91.7)	63 (80.8)	0.056	0.058
**Fat-free mass, kg** (Male *n*: 323)	63.3 ± 14.5	42.5 ± 10.2	60.3 ± 10.4	44.0 ± 8.1	**0.004**	0.491
**Body fat mass, kg** (Male *n*: 322)	20.5 ± 10.5	24.5 ± 9.8	22.1 ± 10.2	26.4 ± 9.9	0.163	0.448
**Body fat percentage (Male *n*: 322)**	22.6 ± 7.5	34.0 ± 7.5	25.4 ± 7.6	35.7 ± 7.2	**0.007**	0.403
(Male ≥ 25%; Female ≥ 32%	28 (11.0)	25 (83.3)	14 (20.6)	74 (94.9)	**0.038**	0.052
**Fat-Free Mass Index** (Male *n*: 322)	21.4 ± 2.1	17.3 ± 1.8	20.6 ± 2.0	17.4 ± 1.7	**0.004**	0.586
**Fat Mass Index** (Male *n*: 322)	6.6 ± 3.2	9.6 ± 3.7	7.5 ± 3.4	10.1 ± 4.0	**0.049**	0.942
**A Body Shape Index**	0.082 ± 0.006	0.077 ± 0.006	0.084 ± 0.004	0.079 ± 0.005	**0.003**	0.384
**Body Adiposity Index**	27.1 ± 3.6	33.7 ± 5.7	28.4 ± 3.9	34.4 ± 5.5	**0.012**	0.403
**ConI**	1.31 ± 0.1	1.22 ± 0.1	1.33 ± 0.1	1.25 ± 0.1	**0.042**	0.381
**Weight-Adjusted Waist Index**	10.8 ± 0.9	10.5 ± 1.1	11.0 ± 0.7	10.7 ± 0.8	**0.001**	0.474
**Abdominal Volume Index**	20.7 ± 6.9	16.0 ± 4.9	20.6 ± 5.3	17.1 ± 4.7	0.773	0.238
**Hip Index**	113.5 ± 16.8	100.7 ± 19.0	110.7 ± 17.5	104.0 ± 16.6	0.335	0.296
**CAP, dB/m** (Male *n*: 325; Female *n*: 107)	249.5 ± 53.2	224.9 ± 46.1	256.5 ± 51.9	230.5 ± 43.9	0.351	0.593
No hepatic steatosis (<248) (*n*, %)	134 (52.3)	20 (66.7)	30 (43.5)	54 (70.1)	0.191	0.728
Hepatic steatosis (≥248) (*n*, %)	122 (47.7)	10 (33.3)	39 (56.5)	23 (29.9)		
**CAP quartiles, dB/m** (*n*, %) (Male *n*: 325; Female *n*: 107)						
Quartile 1 (<211)	55 (21.5)	13 (43.3)	14 (20.3)	25 (32.5)	0.571	0.53
Quartile 2 (211–239)	67 (26.2)	5 (16.7)	13 (18.8)	23 (29.9)		
Quartile 3 (240–277)	60 (23.4)	8 (26.7)	18 (26.1)	20 (26.0)		
Quartile 4 (≥278)	74 (28.9)	4 (13.3)	24 (34.8)	9 (11.7)		
**Probe (XL)**	24 (9.4)	2 (6.7)	6 (8.3)	10 (12.8)	0.863	0.352
**LSM, kPa** (Male *n*: 324; Female *n*: 107)	5.6 ± 1.4	4.5 ± 1.3	5.7 ± 1.3	4.6 ± 1.0	0.964	0.931
No fibrosis (<7) (*n* %)	218 (85.5)	29 (96.7)	54 (78.3)	75 (97.4)	0.342	0.836
Mild–moderate fibrosis (7–9.4) (*n*, %)	35 (13.7)	1 (3.3)	14 (20.3)	2 (2.6)		
Advanced fibrosis (9.5–12.4) (*n*, %)	2 (0.8)	0 (0.0)	1 (1.4)	0 (0.0)		
Cirrhosis (12.5–14.0) (*n*, %)	0 (0.0)	0 (0.0)	0 (0.0)	0 (0.0)		
Within-examination CAP IQR, dB/m	13.1 ± 7.2	15.1 ± 6.9	14.7 ± 6.9	15.2 ± 7.5	0.054	0.970
Within-examination CAP SD, dB/m	10.2 ± 5.0	8.8 ± 4.6	10.0 ± 5.6	9.3 ± 5.0	0.618	0.621

The chi-square test was used for categorical variables, presented as *n* (%), whereas the Mann–Whitney U test was used for continuous variables, presented as mean ± standard deviation (SD). The final two rows describe the within-examination variability of the CAP measurements: the interquartile range (IQR) and the standard deviation (SD) of the valid readings obtained within each examination, both expressed in dB/m. P1 represents the comparison between shift workers and non-shift workers in men, whereas P2 represents the comparison between shift workers and non-shift workers in women. Statistically significant *p*-values (*p* < 0.05) are shown in bold. The sample size varied slightly across variables because of missing data. Abbreviations: CAP, Controlled Attenuation Parameter; LSM, Liver Stiffness Measurement; IQR, Interquartile Range; SD, Standard Deviation.

**Table 3:** Crude Spearman correlations and rank-based adjusted partial Spearman correlations of CAP with anthropometric, adiposity, circadian-rhythm, and sleep-related variables.

Variable	Crude *r_s_* (*p*; *q*)	Model 1 *r_s_* (*p*; *q*)	Model 2 *r_s_* (*p*; *q*)	Model 3 *r_s_* (*p*; *q*)	Model 4 *r_s_* (*p*; *q*)
**Anthropometric and adiposity variables**					
Body Mass Index	0.67 (<0.001; <0.001)	0.65 (<0.001; <0.001)	0.65 (<0.001; <0.001)	0.66 (<0.001; <0.001)	0.66 (<0.001; <0.001)
Waist circumference	0.68 (<0.001; <0.001)	0.65 (<0.001; <0.001)	0.65 (<0.001; <0.001)	0.66 (<0.001; <0.001)	0.66 (<0.001; <0.001)
Neck circumference	0.61 (<0.001; <0.001)	0.58 (<0.001; <0.001)	0.58 (<0.001; <0.001)	0.59 (<0.001; <0.001)	0.60 (<0.001; <0.001)
Waist-to-height ratio	0.66 (<0.001; <0.001)	0.63 (<0.001; <0.001)	0.63 (<0.001; <0.001)	0.63 (<0.001; <0.001)	0.65(<0.001; <0.001)
Waist-to-hip ratio	0.58 (<0.001; <0.001)	0.54 (<0.001; <0.001)	0.54 (<0.001; <0.001)	0.54 (<0.001; <0.001)	0.58 (<0.001; <0.001)
Body fat mass (kg)	0.59 (<0.001; <0.001)	0.65 (<0.001; <0.001)	0.65 (<0.001; <0.001)	0.66 (<0.001; <0.001)	0.66 (<0.001; <0.001)
Fat-free mass (kg)	0.38 (<0.001; <0.001)	0.31 (<0.001; <0.001)	0.31 (<0.001; <0.001)	0.33 (<0.001; <0.001)	0.33 (<0.001; <0.001)
FFMI	0.51 (<0.001; <0.001)	0.48 (<0.001; <0.001)	0.48 (<0.001; <0.001)	0.49 (<0.001; <0.001)	0.49 (<0.001; <0.001)
FMI	0.51 (<0.001; <0.001)	0.65 (<0.001; <0.001)	0.62 (<0.001; <0.001)	0.63 (<0.001; <0.001)	0.63 (<0.001; <0.001)
WWI	0.45 (<0.001; <0.001)	0.39 (<0.001; <0.001)	0.39 (<0.001; <0.001)	0.43 (<0.001; <0.001)	0.43 (<0.001; <0.001)
ConI	0.51 (<0.001; <0.001)	0.44 (<0.001; <0.001)	0.44 (<0.001; <0.001)	0.47 (<0.001; <0.001)	0.47 (<0.001; <0.001)
BAI	0.30 (<0.001; <0.001)	0.46 (<0.001; <0.001)	0.46 (<0.001; <0.001)	0.47 (<0.001; <0.001)	0.47 (<0.001; <0.001)
AVI	0.68 (<0.001; <0.001)	0.65 (<0.001; <0.001)	0.65 (<0.001; <0.001)	0.66 (<0.001; <0.001)	0.66 (<0.001; <0.001)
HI	0.58 (<0.001; <0.001)	0.56 (<0.001; <0.001)	0.56 (<0.001; <0.001)	0.56 (<0.001; <0.001)	0.56 (<0.001; <0.001)
ABSI	0.23 (<0.001; <0.001)	0.12 (0.014; 0.020)	0.12 (0.016; 0.023)	0.14 (0.007; 0.010)	0.14 (0.008; 0.011)
**Circadian rhythm-related variables**					
Social jetlag	−0.01 (0.785; 0.898)	0.05 (0.303; 0.370)	0.05 (0.300; 0.366)	0.06 (0.203; 0.263)	0.07 (0.195; 0.252)
Eating jetlag	−0.01 (0.857; 0.898)	0.05 (0.348; 0.403)	0.04 (0.382; 0.442)	0.05 (0.372; 0.409)	0.05 (0.354; 0.390)
Breakfast jetlag	0.01 (0.825; 0.898)	0.07 (0.198; 0.256)	0.06 (0.205; 0.266)	0.08 (0.142; 0.196)	0.08 (0.130; 0.179)
Lunch jetlag	−0.02 (0.707; 0.865)	0.04 (0.471; 0.518)	0.04 (0.509; 0.555)	0.03 (0.628; 0.658)	0.03 (0.591; 0.619)
Dinner jetlag	−0.04 (0.465; 0.602)	−0.00 (0.993; 0.993)	−0.00 (0.971; 0.971)	−0.00 (0.925; 0.925)	−0.00 (0.948; 0.948)
Sleep duration	0.06 (0.235; 0.323)	0.08 (0.116; 0.160)	0.07 (0.124; 0.171)	0.06 (0.222; 0.272)	0.06 (0.208; 0.254)
PSQI	0.00 (0.921; 0.921)	0.03 (0.537; 0.562)	0.03 (0.530; 0.555)	0.06 (0.254; 0.294)	0.05 (0.274; 0.317)

Spearman rank correlation coefficients were used for the crude model, whereas rank-based partial Spearman correlations were used for the adjusted models; all coefficients are therefore rank-based and do not rely on a normality assumption. Each cell presents Spearman’s r_s_ (raw *p*-value; BH-adjusted *q*-value). Crude model: Spearman correlation analysis without adjustment. Model 1: Partial correlation adjusted for age and sex. Model 2: Partial correlation adjusted for age, sex, and shift-work status. Model 3: Partial correlation adjusted for age, sex, shift-work status, physical activity level (PAL), smoking status, education level, marital status, and income status. Model 4: Partial correlation adjusted for age, sex, shift-work status, physical activity level (PAL), smoking status, education level, marital status, income status, and meal-skipping status. The number of observations differed slightly between variable pairs because pairwise deletion was applied. The Benjamini–Hochberg procedure was applied separately within each model (22 tests per family), and statistical significance after correction for multiple testing was defined as *q* < 0.05. Abbreviations: ABSI, A Body Shape Index; AVI, Abdominal Volume Index; BAI, Body Adiposity Index; BMI, Body Mass Index; CAP, Controlled Attenuation Parameter; ConI, Conicity Index; FFMI, Fat-Free Mass Index; FMI, Fat Mass Index; HI, Hip Index; PAL, Physical Activity Level; PSQI, Pittsburgh Sleep Quality Index; WWI, Weight-Adjusted Waist Index.

**Table 4:** Fully adjusted and simplified multivariable linear regression models for CAP.

Variables	B (95% CI)	SE	β (95% CI)	t	*p*	VIF
**Model A (Fully Adjusted)**						
Age	0.65 (0.13 to 1.17)	0.26	0.12 (0.02 to 0.22)	2.46	**0.014**	1.29
Body Mass Index	3.21 (0.44 to 5.98)	1.41	0.31 (0.04 to 0.58)	2.28	**0.023**	3.60
Waist circumference	0.29 (−0.28 to 0.87)	0.29	0.08 (−0.08 to 0.25)	1.00	0.319	2.19
Neck circumference	3.98 (2.12 to 5.85)	0.95	0.29 (0.16 to 0.43)	4.20	**<0.001**	1.84
Body fat mass (kg)	0.25 (−1.01 to 1.51)	0.64	0.05 (−0.20 to 0.31)	0.39	0.693	3.41
Fat-free mass (kg)	−0.05 (−0.43 to 0.32)	0.19	−0.02 (−0.12 to 0.09)	−0.29	0.775	1.45
Social jet lag	1.17 (−2.86 to 5.20)	2.05	0.02 (−0.06 to 0.11)	0.57	0.568	1.13
Eating jet lag	2.85 (−1.71 to 7.42)	2.32	0.05 (−0.03 to 0.14)	1.23	0.220	1.11
Sleep duration	2.48 (−0.84 to 5.80)	1.69	0.06 (−0.02 to 0.15)	1.47	0.142	1.12
PSQI	−0.59 (−2.14 to 0.95)	0.78	−0.03 (−0.12 to 0.05)	−0.76	0.449	1.11
R = 0.70; R^2^ = 0.49; adjusted R^2^ = 0.45; RMSE = 37.23; F(27, 353) = 12.34; *p* < 0.001; *n* = 381						
**Model B (Simplified)**						
Age	0.54 (0.15 to 0.93)	0.20	0.10 (0.03 to 0.17)	2.74	**0.006**	1.05
Body Mass Index	4.13 (3.13 to 5.12)	0.50	0.40 (0.30 to 0.50)	8.18	**<0.001**	1.80
Neck circumference	4.12 (2.83 to 5.41)	0.66	0.30 (0.21 to 0.40)	6.26	**<0.001**	1.85
R = 0.67; R^2^ = 0.45; adjusted R^2^ = 0.44; RMSE = 38.47; F(3, 428) = 115.05; *p* < 0.001; *n* = 432						

Model A represents the fully adjusted multivariable linear regression model and was adjusted for sex, shift-work status, physical activity level, smoking status, education level, marital status, income status, and meal-skipping behavior. Model B represents the simplified model including only the independent predictors identified in Model A. Multicollinearity was assessed using variance inflation factor (VIF). Statistical significance was defined as *p* < 0.05. Abbreviations: B, Unstandardized Regression Coefficient; CI, Confidence Interval; SE, Standard Error; β, Standardized Regression Coefficient; VIF, Variance Inflation Factor; CAP, Controlled Attenuation Parameter; PSQI, Pittsburgh Sleep Quality Index.

**Table 5:** Sex-specific receiver operating characteristic analyses of anthropometric measurements, body composition parameters, adiposity indices, and circadian rhythm- and sleep-related variables for identifying hepatic steatosis defined as CAP ≥ 248 dB/m.

Variable	AUC	95% CI	Cut-Off	Sensitivity (%)	Specificity (%)	Raw *p*/BH-Adjusted q
**Male**						
Age, years	0.67	0.62–0.73	35.5	73.3	56.1	**<0.001/<0.001**
Body weight, kg	0.83	0.79–0.87	80.45	87.0	63.4	**<0.001/<0.001**
BMI, kg/m^2^	0.85	0.80–0.89	27.75	77.6	76.8	**<0.001/<0.001**
Waist circumference, cm	0.86	0.82–0.90	102.2	69.6	85.4	**<0.001/<0.001**
Neck circumference, cm	0.82	0.77–0.86	40.05	70.8	77.4	**<0.001/<0.001**
Body fat mass, kg	0.84	0.80–0.88	18.19	83.2	72.5	**<0.001/<0.001**
Fat-free mass, kg	0.69	0.64–0.75	69.05	43.5	86.8	**<0.001/<0.001**
FFMI	0.77	0.71–0.82	20.95	75.7	69.0	**<0.001/<0.001**
FMI	0.83	0.78–0.88	5.75	84.5	66.2	**<0.001/<0.001**
Waist-to-height ratio	0.84	0.80–0.88	0.557	83.9	67.7	**<0.001/<0.001**
Waist-to-hip ratio	0.82	0.77–0.87	0.964	64.0	86.6	**<0.001/<0.001**
ConI	0.78	0.73–0.83	1.304	80.8	68.9	**<0.001/<0.001**
WWI	0.75	0.70–0.81	10.91	64.6	76.2	**<0.001/<0.001**
BAI	0.75	0.70–0.81	26.12	83.9	57.3	**<0.001/<0.001**
AVI	0.86	0.82–0.90	20.97	69.6	85.4	**<0.001/<0.001**
HI	0.80	0.75–0.85	112.53	73.9	73.8	**<0.001/<0.001**
ABSI	0.62	0.56–0.68	0.0816	67.7	53.1	**<0.001/<0.001**
Social jet lag, h	0.49	0.43–0.55	2.125	9.8	91.9	0.735/0.735
Eating jet lag, h	0.52	0.46–0.58	0.75	36.0	70.7	0.442/0.464
Sleep duration, h	0.53	0.46–0.59	6.25	77.0	31.9	0.406/0.449
PSQI	0.47	0.41–0.53	1.5	94.4	9.2	0.382/0.447
**Female**						
Age, years	0.59	0.47–0.72	48.5	30.3	90.5	0.124/0.137
Body weight, kg	0.78	0.70–0.87	65.25	96.9	56.2	**<0.001/<0.001**
BMI, kg/m^2^	0.81	0.73–0.89	27.0	84.9	67.6	**<0.001/<0.001**
Waist circumference, cm	0.77	0.69–0.86	84.5	100.0	46.0	**<0.001/<0.001**
Neck circumference, cm	0.73	0.63–0.82	33.75	100.0	41.9	**<0.001/<0.001**
Body fat mass, kg	0.79	0.71–0.88	23.55	87.9	66.2	**<0.001/<0.001**
Fat-free mass, kg	0.62	0.50–0.74	46.15	63.6	64.9	0.050/0.066
FFMI	0.75	0.66–0.85	16.75	96.7	47.1	**<0.001/<0.001**
FMI	0.79	0.71–0.88	8.45	93.3	57.1	**<0.001/<0.001**
Waist-to-height ratio	0.79	0.70–0.87	0.555	84.9	67.6	**<0.001/<0.001**
Waist-to-hip ratio	0.69	0.59–0.79	0.830	87.9	55.4	**0.001/0.002**
ConI	0.65	0.54–0.76	1.295	50.0	76.7	**0.015/0.023**
WWI	0.67	0.56–0.78	10.44	87.5	52.1	**0.006/0.009**
BAI	0.77	0.68–0.86	35.20	69.7	78.4	**<0.001/<0.001**
AVI	0.78	0.69–0.86	15.28	90.9	56.8	**<0.001/<0.001**
HI	0.75	0.65–0.84	98.01	84.4	56.2	**<0.001/<0.001**
ABSI	0.46	0.34–0.57	0.0824	84.9	23.0	0.500/0.525
Social jet lag, h	0.59	0.48–0.71	1.10	54.6	72.6	0.115/0.135
Eating jet lag, h	0.47	0.34–0.59	1.575	21.2	88.7	0.583/0.583
Sleep duration, h	0.63	0.52–0.74	7.75	57.6	62.2	**0.030/0.042**
PSQI	0.61	0.50–0.73	6.5	36.4	86.5	0.057/0.071

Hepatic steatosis was defined as CAP ≥ 248 dB/m. Receiver operating characteristic (ROC) analyses were performed separately for women and men. Optimal cut-off values were determined using Youden’s index. Sensitivity and specificity were calculated according to the selected cut-off values. The final column presents raw *p*/BH-adjusted q. The Benjamini–Hochberg procedure was applied separately within each sex (21 tests per family). Abbreviations: AUC, area under the receiver operating characteristic curve; CI, confidence interval; CAP, controlled attenuation parameter; ConI, conicity index; BMI, body mass index; FFMI, fat-free mass index; FMI, fat mass index; BAI, body adiposity index; AVI, abdominal volume index; WWI, weight-adjusted waist index; ABSI, a body shape index; HI, hip index; PSQI, Pittsburgh Sleep Quality Index.

## Data Availability

The data supporting the findings of this study are available from the corresponding author upon reasonable request.

## Abbreviations

The following abbreviations are used in this manuscript:

ABSI	A body shape index
AUC	Area under the curve
AVI	Abdominal volume index
β	Standardized coefficient
BAI	Body adiposity index
BIA	Bioelectrical impedance analysis
BMI	Body mass index
CAP	Controlled attenuation parameter
CI	Confidence interval
ConI	Conicity index
EJL	Eating jetlag
FDR	False discovery rate
FFM	Fat-free mass
FFMI	Fat-free mass index
FMI	Fat mass index
HC	Hip circumference
HI	Hip index
IQR	Interquartile range
LSM	Liver stiffness measurement
MASH	Metabolic dysfunction-associated steatohepatitis
MASLD	Metabolic dysfunction-associated steatotic liver disease
NAFLD	Non-alcoholic fatty liver disease
NC	Neck circumference
PAL	Physical activity level
PSQI	Pittsburgh Sleep Quality Index
RMSE	Root mean square error
ROC	Receiver operating characteristic
SD	Standard deviation
SE	Standard error
SJL	Social jetlag
VIF	Variance inflation factor
WC	Waist circumference
WHR	Waist-to-hip ratio
WHtR	Waist-to-height ratio
WWI	Weight-adjusted waist index
